# NADH oxidase of *Mycoplasma synoviae* is a potential diagnostic antigen, plasminogen/fibronectin binding protein and a putative adhesin

**DOI:** 10.1186/s12917-022-03556-2

**Published:** 2022-12-29

**Authors:** Zengjin Hu, Haoran Li, Yuxin Zhao, Guijun Wang, Yuanbing Shang, Yuetong Chen, Shaohui Wang, Mingxing Tian, Jingjing Qi, Shengqing Yu

**Affiliations:** 1 Shanghai Veterinary Research Institute, the Chinese Academy of Agricultural Sciences (CAAS), 518 Ziyue Road, Shanghai, 200241 People’s Republic of China; 2 College of Animal Science and Technology, Anhui Agricultural University, No. 130 Changjiangxilu, Hefei, Anhui 230061 People’s Republic of China; 3 College of Veterinary Medicine, Yangzhou University, No. 88 University South Road, Yangzhou, Jiangsu 225009 People’s Republic of China

**Keywords:** *Mycoplasma synoviae*, NADH oxidase, Membrane-localization, Diagnostic antigen, Cytoadherence, Plasminogen/fibronectin-binding

## Abstract

**Background:**

*Mycoplasma synoviae* (MS) is an important pathogen causing respiratory diseases and arthritis in chickens and turkeys, thus, resulting in serious economic losses to the poultry industry. Membrane-associated proteins are thought to play important roles in cytoadherence and pathogenesis. NADH oxidase (NOX) is an oxidoreductase involved in glycolysis, which is thought to be a multifunctional protein and potential virulence factor in some pathogens. However, little is known regarding the NOX of MS (MSNOX). We previously demonstrated that MSNOX was a metabolic enzyme distributed in not only the cytoplasm but also the MS membrane. This study was aimed at exploring NOX’s potential as a diagnostic antigen and its role in MS cytoadherence.

**Results:**

Western blots and ELISAs indicated that recombinant MSNOX (rMSNOX) protein reacted with sera positive for various MS isolates, but not MG isolates or other avian pathogens, thus, suggesting that rMSNOX is a potential diagnostic antigen. In addition, rabbit anti-rMSNOX serum showed substantial complement-dependent mycoplasmacidal activity toward various MS isolates and MG R_low_. MSNOX protein was found not only in the cytoplasm but also on the membrane of MS through suspension immunofluorescence and immunogold electron microscopy assays. Indirect immunofluorescence assays indicated that rMSNOX adhered to DF-1 cells, and this adherence was inhibited by rabbit anti-rMSNOX, but not anti-MG serum. Furthermore, indirect immunofluorescence and colony counting assays confirmed that the rabbit anti-rMSNOX serum inhibited the adherence of various MS isolates but not MG R_low_ to DF-1 cells. Moreover, plasminogen (Plg)- and fibronectin (Fn)-binding assays demonstrated that rMSNOX bound Plg and Fn in a dose-dependent manner, thereby further confirming that MSNOX may be a putative adhesin.

**Conclusion:**

MSNOX was identified to be a surface immunogenic protein that has good immunoreactivity and specificity in Western blot and ELISA, and therefore, may be used as a potential diagnostic antigen in the future. In addition, rMSNOX adhered to DF-1 cells, an effect inhibited by rabbit anti-rMSNOX, but not anti-MG serum, and anti-rMSNOX serum inhibited the adherence of various MS isolates, but not MG R_low_, to DF-1 cells, thus indicating that the inhibition of adherence by anti-MSNOX serum was MS specific. Moreover, rMSNOX adhered to extracellular matrix proteins including Plg and Fn, thus suggesting that NOX may play important roles in MS cytoadherence and pathogenesis. Besides, rabbit anti-rMSNOX serum presented complement-dependent mycoplasmacidal activity toward both MS and MG, indicating the MSNOX may be further studied as a potential protective vaccine candidate.

**Supplementary Information:**

The online version contains supplementary material available at 10.1186/s12917-022-03556-2.

## Background

*Mycoplasma synoviae* (MS) causes infectious synovitis, respiratory disease, egg production losses, and eggshell abnormalities in the commercial poultry industry [1]. MS is usually considered to cause subclinical upper respiratory infection. However, when combined with Newcastle disease or infectious bronchitis, MS can lead to respiratory conditions with air sac disease or infectious synovitis after systemic progression [2]. MS infections have been reported worldwide, and epidemiological surveillance has revealed that MS has a high prevalence rate in commercial chicken flocks [3–5]. Although the disease is rarely associated with mortality, it can cause substantial economic losses to the poultry industry. In China, the seroprevalence of MS among 44,395 non-vaccinated chickens from 21 provinces from 2010 to 2015 has been reported to be 41.19% [6], thus indicating that MS infection is widespread in China. The pathogenesis of MS must be clarified to aid in further development of diagnostic antigens, subunit vaccines, and therapeutic drugs against MS infections.

Adherence is the initial step in which a pathogen colonizes and infects host cells. Cytoadherence-associated proteins may play important roles in pathogenesis. Owing to a lack of cell walls, the adherence of mycoplasma to host cells depends primarily on surface membrane-associated proteins [7, 8]. The most studied MS adhesin is variable lipoprotein hemagglutinin (VlhA), a highly divergent virulence-associated factor [9–11]. In mycoplasmas, some glycolytic enzymes, such as enolase [12–15], glyceraldehyde-3-phosphate dehydrogenase (GAPDH) [16], and the pyruvate dehydrogenase alpha and beta subunits (PdhA and PdhB) [17] have been identified as “moonlighting proteins”, which not only function as metabolic enzymes in the cytoplasm but also are displayed on the pathogen surface, and bind host cells or host components.

NADH oxidase (NOX) in bacteria catalyzes the oxidation of NADH to NAD^+^ by simultaneously reducing O_2_ to H_2_O or H_2_O_2_ [18, 19]. In *Streptococcus*, deletion or mutation of the *nox* gene affects bacterial growth, biofilm formation, competitiveness, and virulence [20–23], thus, suggesting that NOX is important in multiple biological functions. *Mycoplasma bovis* (*M. bovis*) NOX has been demonstrated to function as both an active NADH oxidase and an adhesin, and is therefore a potential virulence factor [24]. NOX has been identified as a major immunogenic protein of MS through electrophoresis and western blotting combined with N-terminal sequencing [25]. In our previous study, we confirmed that MSNOX could oxidize NADH to NAD^+^, and was distributed in both the cytoplasm and membrane components of MS [26]. The membrane localization of MSNOX protein suggests that it may participate in the interaction between MS and cell hosts. In this study, we further confirmed that NOX was surface localized on MS cells, and bound host cells and extracellular matrix (ECM) proteins, including plasminogen (Plg) and fibronectin (Fn). Moreover, rabbit anti-rMSNOX serum exhibited significant adherence inhibition activity. These results further indicated that MSNOX plays an important role in cytoadherence.

## Results

### Expression and immunological analysis of rMSNOX

**Fig. 1:**
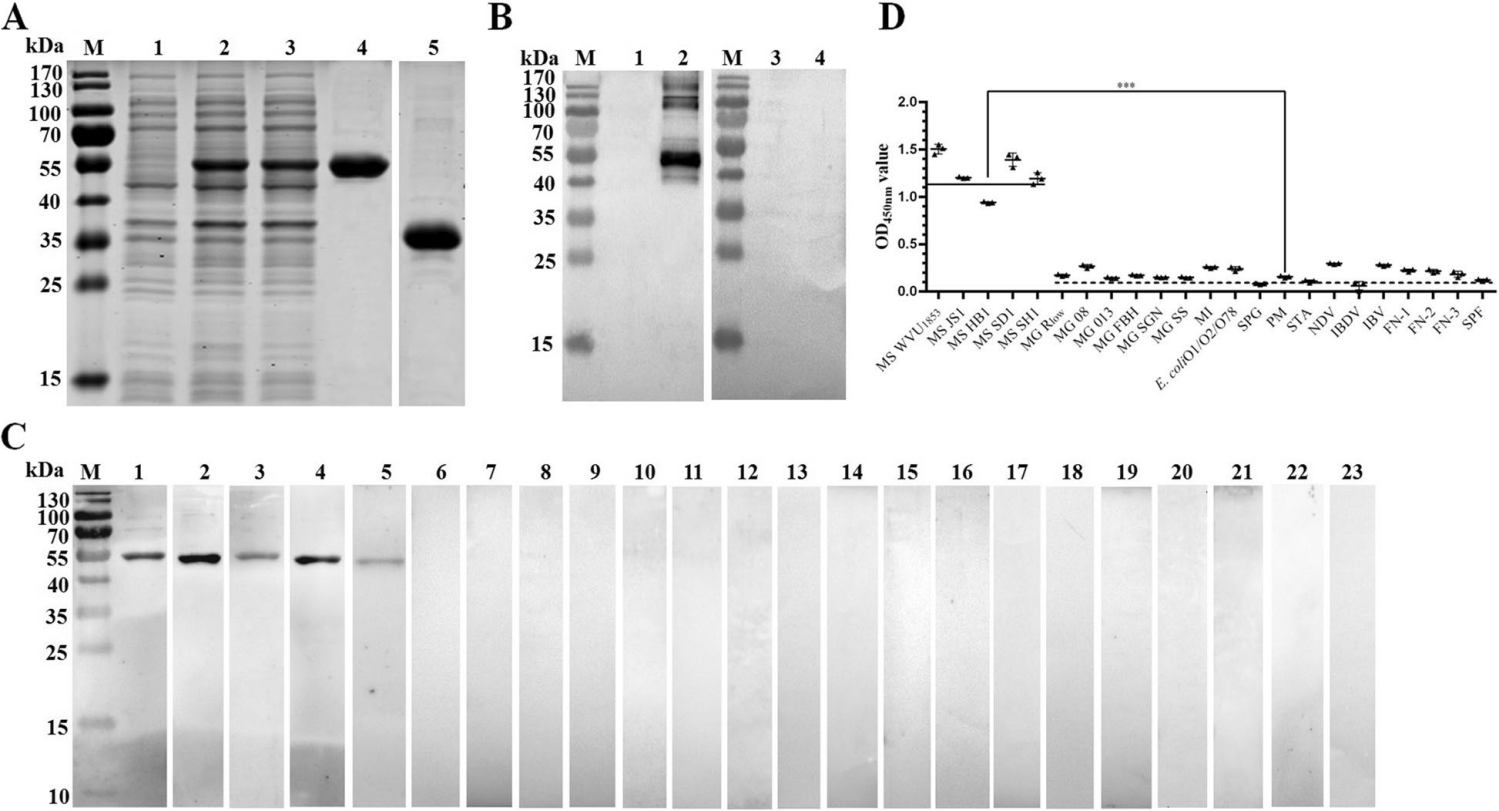
Expression and immunological characteristics of rMSNOX protein. **A** SDS-PAGE analysis of expression and purification of rMSNOX protein. Lane M: protein ladder; lane 1: cell lysates of *E. coli* BL21 containing empty vector; lane 2: total cell lysates of recombinant strain *E. coli* BL21 (pET28a-MS*nox*); lane 3: supernatant of total cell lysates of recombinant bacteria; lane 4: purified His-tagged MSNOX protein; lane 5: purified His-tagged MSFBA protein. The gels in this figure were cropped, and the original gel is presented in Supplementary Fig. S2. **B** Immunogenicity analysis of rMSNOX protein. Lanes1 and 2: western blot analysis of the purified rMSFBA and rMSNOX with rabbit anti-rMSNOX serum; lane 3 and 4: western blot analysis of the purified rMSFBA and rMSNOX with pre-immune rabbit serum. The blots were cropped from two blots, which are presented in Supplementary Fig. S3. Immunoreactivity and specificity analysis of rMSNOX, on the basis of western blot (**C**) and ELISAs (**D**). Lanes 1–5: chicken sera positive for various MS isolates (MS WVU_1853_, MS JS1, MS HB1, MS SD1, and MS SH1); lanes 6–11: chicken sera positive for various MG isolates (MG R_low_, MG 08, MG 013, MG FBH, MG SGN, and MG SS); lanes 12–19: sera positive for other avian pathogens (MI, *E. coli* O1/O2/O78, SPG, PM, STA, NDV, IBDV, and IBV); lane 20–22: field MS-negative sera (FN-1, FN-2, and FN-3); 23: negative serum from SPF chickens. Blots were cropped from different gels and are divided by white space (Supplementary Fig. S4). The samples derived from western blotting experiments, and the gels/blots were processed in parallel. Significant differences between OD_450 nm_ values of chicken sera positive for various MS isolates and other avian pathogens were analyzed with unpaired T-test in GraphPad Prism 6

His-tagged recombinant MSNOX (rMSNOX) protein and recombinant MSFBA (rMSFBA) protein (His-tag control) were expressed in *E. coli* BL21 and purified. The purified rMSNOX and rMSFBA proteins were analyzed with sodium dodecyl sulfate polyacrylamide gel electrophoresis (SDS-PAGE) (Fig. 1A). Rabbit anti-rMSNOX, anti-rMSFBA, anti-MS, and anti-MG sera were prepared, and their antibody titers were detected to be 1:102,400,1:1:51,200, 1:25,600, and 1:102,400, respectively by ELISAs (Fig. S1). Western blot assays showed that the rMSNOX protein, but not rMSFBA, was recognized by rabbit anti-rMSNOX serum, whereas neither was recognized by pre-immune rabbit serum (Fig. 1B), thus suggesting that MSNOX had good immunogenicity.

The immunoreactivity and specificity of MSNOX was analyzed by western blotting and ELISAs. The results of western blotting showed that the purified rMSNOX protein reacted with chicken sera positive for various MS isolates (Fig. 1C, lanes 1–5), but not various *Mycoplasma gallisepticum* (MG) isolates (Fig. 1C, lanes 6–11). Sera positive for several other avian pathogens including *Mycoplasma iowae* (MI), *Escherichia coli* (*E. coli*) O1/O2/O78, *Salmonella pullorum/gallinarum* (SPG), *Pasteurella multocida* (PM), *Staphylococcus aureus* (STA), Newcastle disease virus (NDV), infectious bursal disease virus (IBDV), and avian infectious bronchitis virus (IBV) (Fig. 1C, lanes 12–19); three field MS-negative chicken sera (FN-1, FN-2, and FN-3; Fig. 1C, lanes 20–22); or specific pathogen free (SPF) chicken serum (Fig. 1C, lane 23) were also tested. The results of ELISAs showed that the OD_450 nm_ values of positive chicken sera of various MS isolates were all above 0.9, whereas the OD_450 nm_ values of chicken sera positive for various MG isolates and other avian pathogens were below 0.3 (Fig. 1D). Significant differences were observed among the sera (^***^p < 0.001). The ELISA results were consistent with the western blot results, thus demonstrating that the rMSNOX had good immunoreactivity and specificity.

### Complement dependent mycoplasmacidal assays

**Fig. 2:**
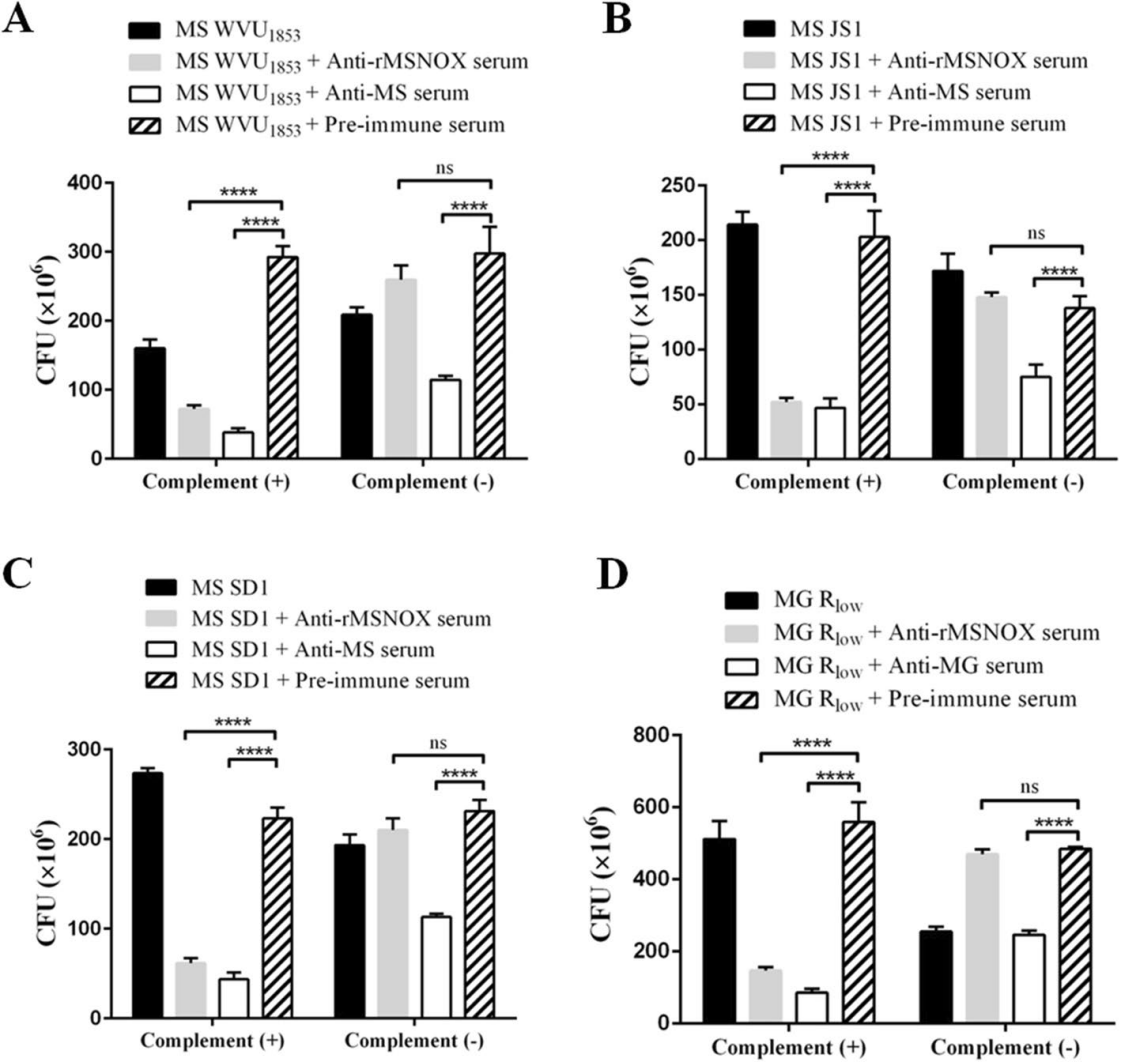
Mycoplasmacidal assays. The mycoplasmacidal activity of rabbit anti-rMSNOX serum toward MS WVU_1853_ (**A**), MS JS1 (**B**), MS SD1 (**C**), or MG R_low_ (**D**) with complement ( +) or without complement (-) was detected. Rabbit anti-MS/MG serum and pre-immune rabbit serum were used as controls. Significant differences between anti-rMSNOX serum and pre-immune serum were analyzed with two-way ANOVA in GraphPad Prism 6

Compared with pre-immune rabbit serum, rabbit anti-rMSNOX serum showed significant mycoplasmacidal activity toward all three MS isolates and MG R_low_ when complement was added (^****^p < 0.0001), but little or no bactericidal effect (ns, no significance) when complement was not added (Fig. 2). The rabbit anti-MS or anti-MG sera showed significant bactericidal effects against various MS strains or MG R_low_ with or without complement. These results indicated that the rabbit anti-rMSNOX serum together with complement had good mycoplasmacidal activity against both MS and MG.

### Surface localization of NOX on MS

**Fig. 3:**
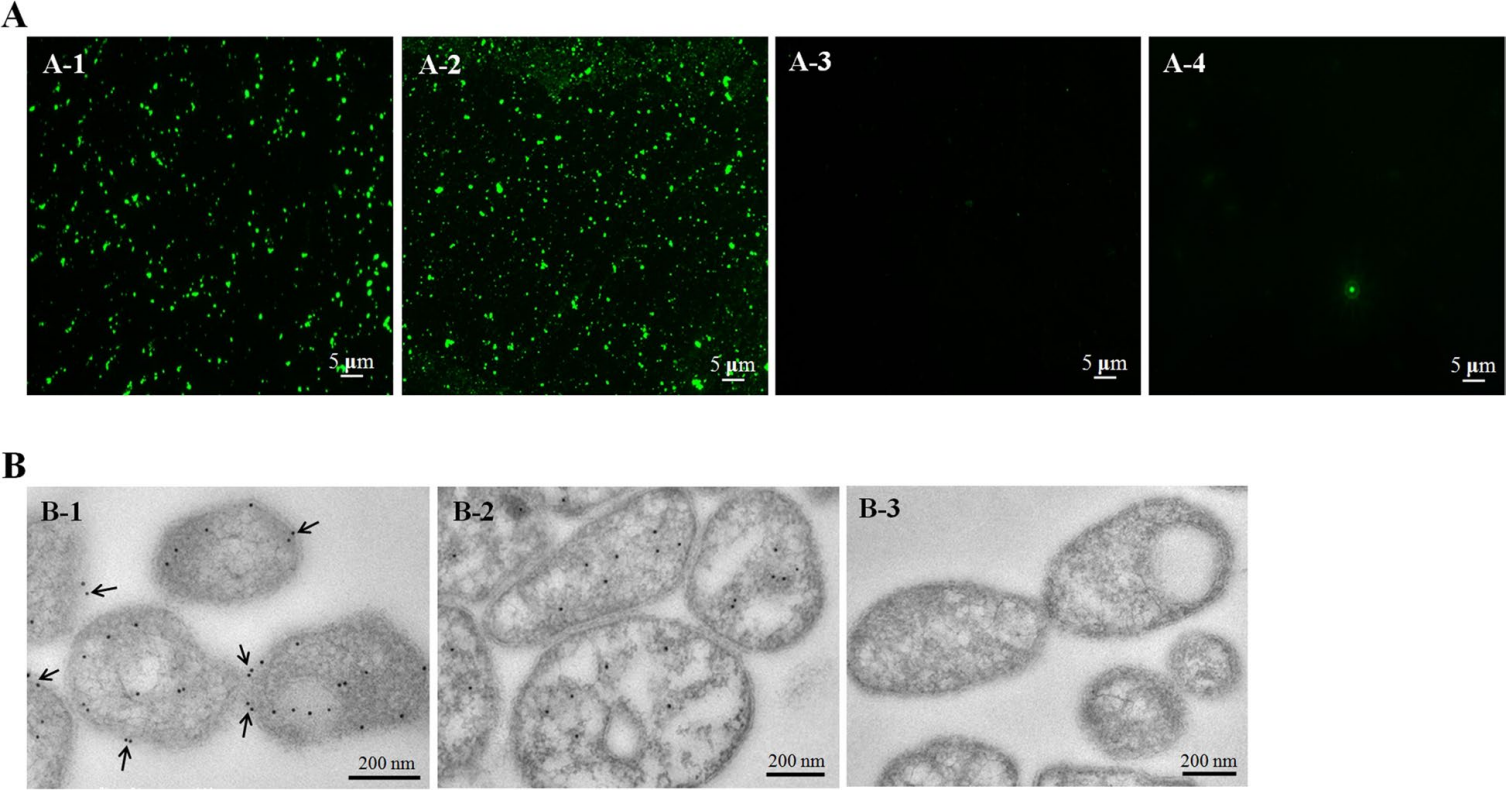
Surface localization analysis of MSNOX. **A** Suspension immunofluorescence assays. MS cells were treated with rabbit anti-rMSNOX serum (A-1), rabbit anti-MS serum (A-2), rabbit anti-rMSFBA serum (A-3), or pre-immune rabbit serum (A-4), followed by goat anti-rabbit IgG-FITC antibody. **B** Immunogold electron transmission microscopy assays. (B-1) Subcellular localization of NOX proteins in MS. Black arrows indicate the membrane localized NOX proteins. (B-2) Subcellular localization of FBA proteinsin MS. (B-3) Pre-immune rabbit serum treated MS cells

**Fig. 4:**
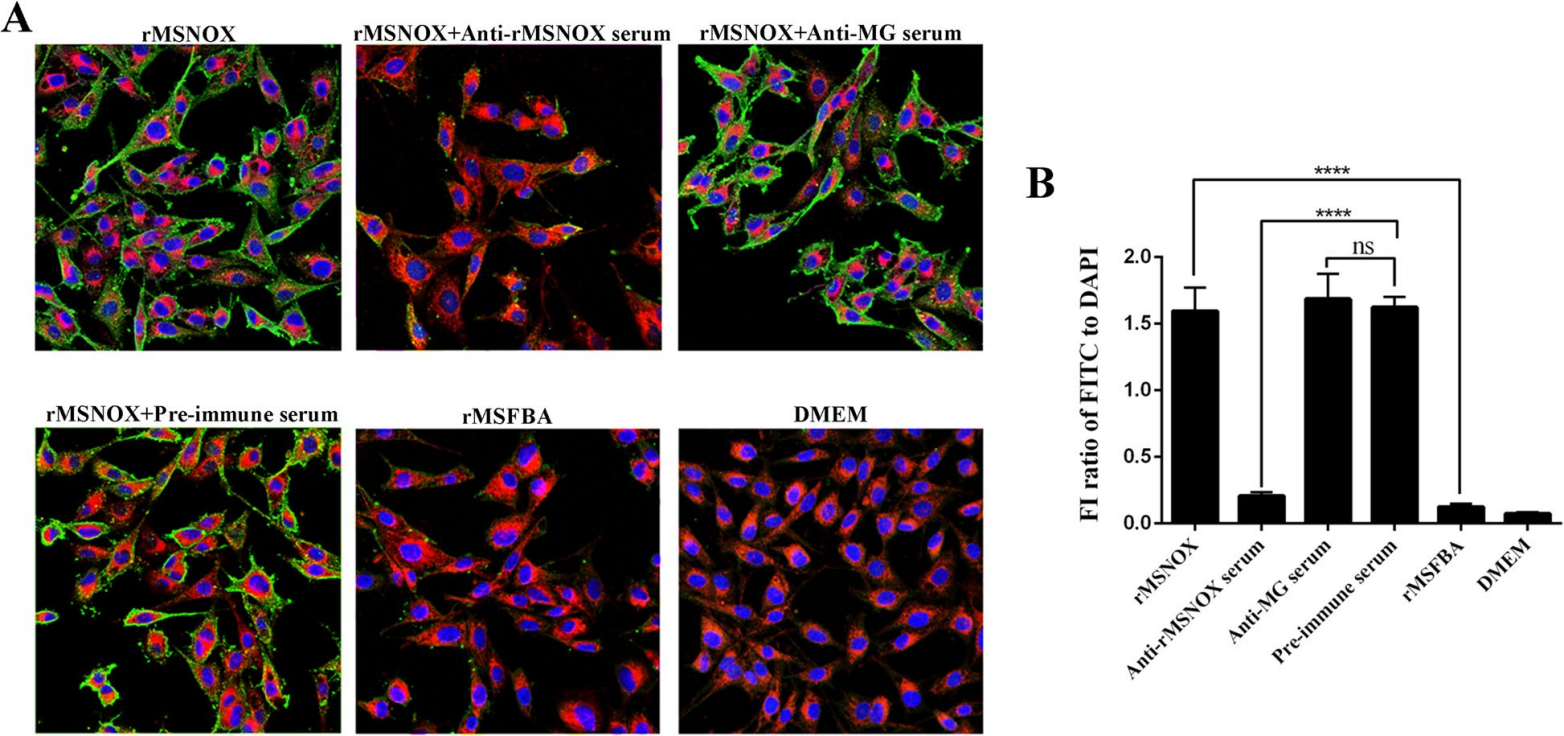
Adherence and inhibition of rMSNOX to DF-1 cells. **A** Adherence of rMSNOX to DF-1 cells were detected by indirect immunofluorescence assays. rMSNOX protein was pre-treated with rabbit anti-rMSNOX, anti-MG, or pre-immune serum, then allowed to adhere to DF-1 cells. rMSFBA protein (His-tagged cytoplasmic protein control) and DMEM (blank control) treated DF-1 cells were also detected. Both rMSNOX and rMSFBA protein were labeled with FITC-conjugated antibodies (green). The DF-1 cell membranes and nuclei were stained with Dil (red) and DAPI (blue). **B** The fluorescence intensity (FI) ratios of FITC (green) to DAPI (blue) for NOX adherence were assessed by ImageJ software. Significant differences were analyzed with one-way ANOVA in GraphPad prism 6

Suspension immunofluorescence assays using rabbit anti-rMSNOX serum or anti-MS serum (positive control) showed green fluorescence on the surface of MS (Fig. 3A-1 and A-2), whereas no green fluorescence was observed when rabbit anti-rMSFBA (Fig. 3A-3, cytoplasmic protein control) or pre-immune rabbit serum (Fig. 3A-4) was used as the primary antibody. These findings demonstrated that MSNOX protein was present on the MS membrane surface.

The distribution of MSNOX and MSFBA was examined through immunogold electron microscopy using rabbit anti-rMSNOX or anti-rMSFBA serum with goat anti-rabbit IgG gold conjugate antibody as probes. The NOX proteins were distributed in both the cytoplasm and membrane of MS cells (Fig. 3B-1), whereas almost all MSFBA proteins were distributed in cytoplasmic components (Fig. 3B-2). MS cells treated with pre-immune rabbit serum with goat anti-rabbit IgG gold conjugate antibody showed no signals in blots (Fig. 3B-3).

### Adherence and inhibition of adherence of rMSNOX protein to DF-1 cells

The adherence of rMSNOX protein to DF-1 cells was identified by indirect immunofluorescence using rabbit anti-rMSNOX serum as the primary antibody and fluorescein isothiocyanate (FITC)-conjugated goat anti-rabbit IgG as the secondary antibody (Fig. 4A). The fluorescence intensity (FI) ratios of FITC (green) to DAPI (blue) of each treatment from three repeat tests were analyzed and shown in Fig. 4B, which help us judge the relative amount of adhered protein. The results indicated that rMSNOX protein adheres to DF-1 cells, the adherence was stronger than that of rMSFBA (^****^p < 0.001), and can be significantly inhibited by anti-rMSNOX serum (^****^p < 0.001), but not affected by rabbit anti-MG serum (ns) when compared with pre-immune rabbit serum. Cells treated with Dulbecco’s modified Eagle’s medium (DMEM) showed very little or no green fluorescence on the surface.

### Adherence and inhibition of adherence of MS or MG to DF-1 cells

Indirect immunofluorescence and colony counting assays were performed to determine the adherence and inhibition of adherence of MS or MG to DF-1 cells.

**Fig. 5:**
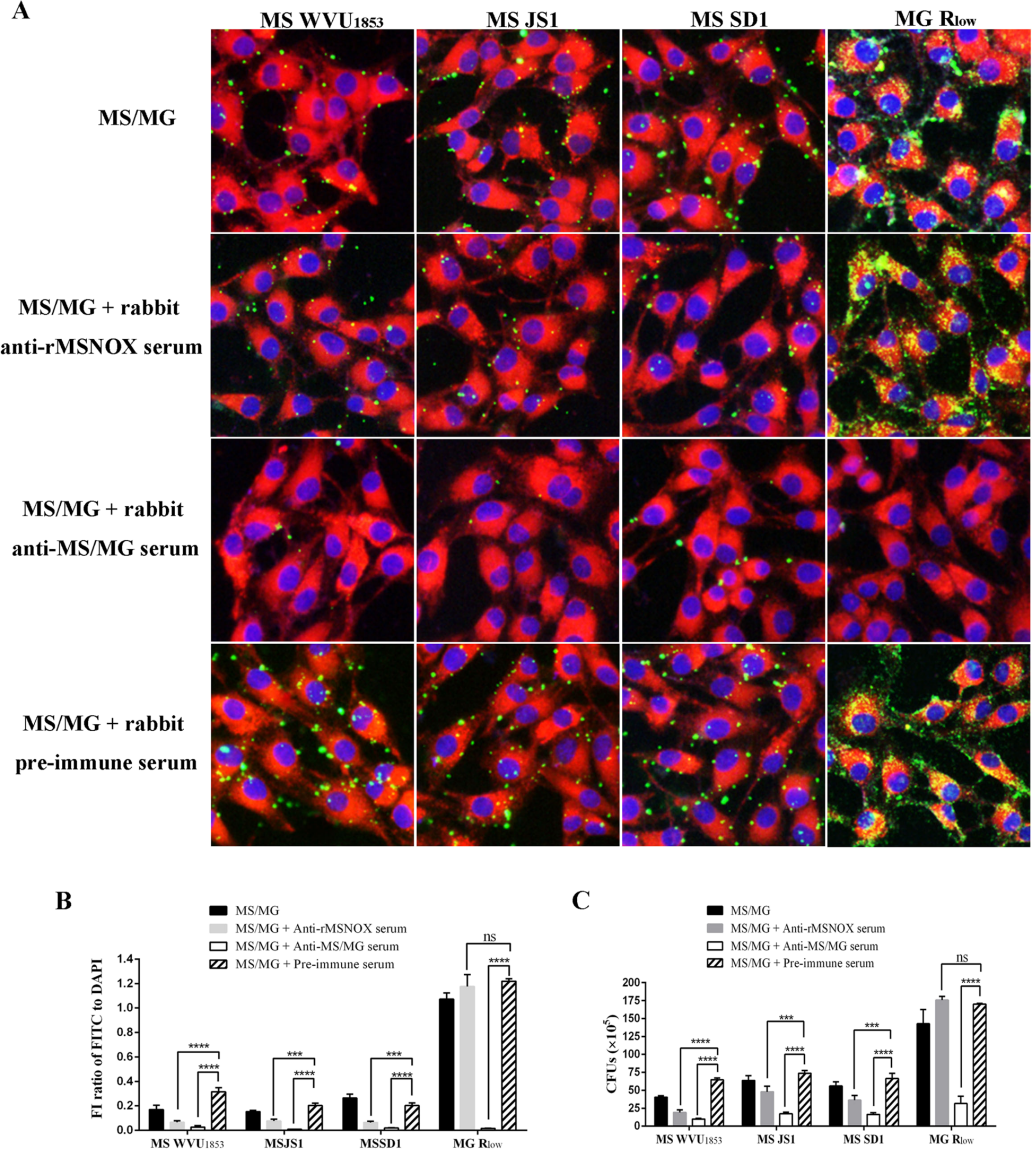
Adherence and inhibition of MS or MG to DF-1 cells. MS WVU_1853_, MS JS1, MS SD1, and MG R_low_ were pre-incubated with rabbit anti-rMSNOX, anti-MS (for MS isolates), anti-MG (for MG R_low_), or pre-immune serum, which were then allowed to adhere to DF-1 cells. In indirect immunofluorescence assays (**A**), the adhered mycoplasma were labeled with goat anti-rabbit IgG-FITC (green), and the cell membranes and nuclei were stained with Dil (red) and DAPI (blue), respectively. **B** The FI ratios of FITC to DAPI for adhered MS or MG bacteria were assessed by ImageJ software. In colony counting assays (**C**), the CFUs of adhered MS or MG bacteria with each treatment were assessed through colony counting. Significant differences were analyzed with two-way ANOVA in GraphPad prism 6

In indirect immunofluorescence assays, the MS or MG bacteria were labeled with rabbit anti-MS or anti-MG serum with FITC-conjugated goat anti-rabbit IgG antibody. The pictures and relative quantitative analysis of FITC/DAPI of each treatment from three repeats are shown in Fig. 5A and B. The results showed that all the mycoplasma, including various MS isolates (WVU_1853_, JS1, and SD1) and MG R_low_, adhered to DF-1 cells, and the number of adherent MG exceeded that of MS. All MS strains (WVU_1853_, JS1, and SD1) were significantly inhibited by rabbit anti-rMSNOX serum or rabbit anti-MS serum (^***^ *p* < 0.01 or ^****^p < 0.001), as compared with pre-immune rabbit serum. Moreover, the MG R_low_ bacteria were significant inhibited by rabbit anti-MG serum (^****^p < 0.001), but not by anti-rMSNOX serum (ns), as compared with pre-immune rabbit serum.

The results of colony counting assays are shown in Fig. 5C. Rabbit anti-rMSNOX or anti-MS serum, compared with pre-immune rabbit serum, significantly inhibited the adherence of MS WVU_1853_, MS JS1, and MS SD1 to DF-1 cells (^***^ *p* < 0.01 or ^****^p < 0.001). Moreover, MG R_low_ adherence was also significantly inhibited by rabbit anti-MG serum (^****^p < 0.001), but not by rabbit anti-rMSNOX serum (ns). These results were consistent with those of indirect immunofluorescence assays. Together, the results indicated that rabbit anti-rMSNOX serum inhibited the adherence of various MS isolates, but not MG R_low_, to DF-1 cells.

### Chicken plasminogen (cPlg) and human fibronectin (hFn) binding ability of rMSNOX

**Fig. 6:**
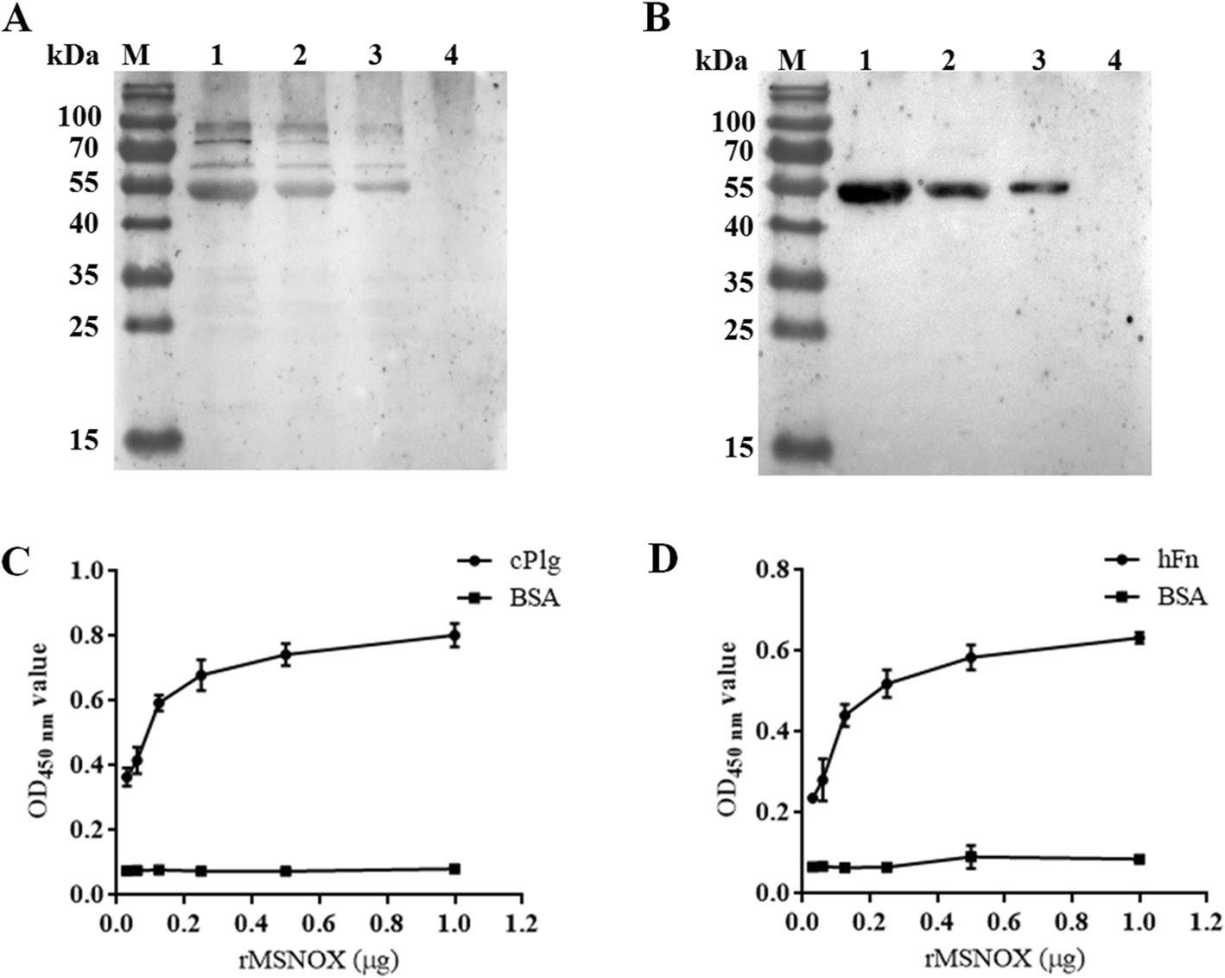
Determination of rMSNOX binding to cPlg and hFn. The binding ability of rMSNOX to cPlg (**A**) and hFn (**B**), confirmed by western blot assays. Gradient diluted rMSNOX protein (lanes 1–3: 2, 1, and 0.5 µg) and 2 µg BSA (lane 4) were incubated with 10 µg/mL of cPlg or hFn, then recognized by rabbit anti-cPlg or anti-hFn polyclonal antibody (1:1000). The full-length figures are presented in Supplementary Fig. S5. Binding ability of rMSNOX to cPlg (**C**) and hFn (**D**),identified by ELISAs with rabbit anti-rMSNOX serum (1:500). Plates were coated with 1 µg of cPlg or hFn, then incubated with serially diluted rMSNOX protein (1, 0.5, 0.25, 0.125, 0.0625, and 0.03125 µg/well). Wells coated with 1 µg of BSA were used as a negative control

The binding ability of rMSNOX to cPlg and hFn was confirmed by western blotting and ELISAs. The western blot assays showed that both cPlg (Fig. 6A) and hFn (Fig. 6B) bound rMSNOX protein with bands of approximately 53 kDa, and the binding ability was dose dependent. No binding band was observed for bovine serum albumin (BSA) with cPlg/hFn under the same conditions. Moreover, the ELISAs were performed with plates coated with cPlg, hFn or BSA, then incubated with serially diluted rMSNOX protein. The results also confirmed that the rMSNOX protein interacted with cPlg (Fig. 6C) and hFn (Fig. 6D) in a dose-dependent manner.

## Discussion

The adherence of pathogenic microorganisms to host cells is an initial step in infection. Because mycoplasmas have no cell walls, membrane proteins play an important role in the interaction between mycoplasma and host, and provide a route for this interaction [27]. The NOX in bacteria usually exists in the cytoplasm, where it plays an important role in regulating cell metabolism, such as maintaining the dynamic balance of NADH/NAD^+^ in glycolysis. In our study, MSNOX was found to be distributed not only in the cytoplasm but also on the membrane of MS, thus suggesting that it may be involved in cytoadherence. In *Streptococcus pneumoniae* (*S. pneumoniae*) [28] and *M. bovis* [24], *nox*-deficient strains show significantly diminished adherence to host cells, thus indicating that NOX may act as an adhesin. Indirect immunofluorescence assays showed that rMSNOX adhered to DF-1 cells, and this adherence was significantly inhibited by rabbit anti-rMSNOX serum but not by rabbit anti-MG serum. Furthermore, both indirect immunofluorescence and colony counting assays indicated that various MS strains (WVU_1853_, JS1 and SD1) adhered to DF-1 cells, and this adherence was inhibited by anti-rMSNOX, suggesting that MSNOX might play an important role in MS cytoadherence. In contrast, both methods confirmed that the adherence of MG R_low_ was inhibited only by rabbit anti-MG serum but not anti-rMSNOX serum, thus indicating that anti-MSNOX serum had an adherence inhibitory effect on only MS but not MG.

Fn and Plg are widely known ECM proteins that are common host cell factors promoting the interaction between pathogens and host cells [29–31]. Bacterial adherence to host tissues and ECM proteins is a critical step in infection, because it establishes the initial contact with the host [32]. Fn plays important roles in several biological processes, such as adherence to ECM, differentiation, growth, and cell migration, and it is considered as the target of many bacterial proteins [33]. The ability to bind Fn is a characteristic reported for many pathogens [34]. Studies of the invasive ability of *Staphylococcus aureus* have suggested that Fn-binding is a major virulence trait that enables this pathogen to invade and cause disease and to persist within host cells [35]. Plg is the pro-enzyme of plasmin, an enzyme in the fibrinolytic system. Some pathogenic microorganisms express proteins that bind and enhance the activity of plasminogen [36–38]. In this way, pathogens use the host fibrinolytic system to promote invasion and colonization of the host [39–42]. In mycoplasma, some adherence-associated proteins have been identified to bind Fn or Plg [12, 17, 43]. In this study, we showed that rMSNOX bound cPlg and hFn in a dose-dependent manner, thereby suggesting that MSNOX may play an important pathogenic role in the adherence and invasion of MS to host cells. In *S. pneumoniae* [44] and *S. suis* [45], NOX was demonstrated to be an essential factor for infection, and the virulence of NOX mutants was significantly diminished. The precise mechanism through which NOX is involved in the pathogenesis of MS remains to be explored. The C-terminal lysine residues of *Mycoplasma hyorhinis* enolase were identified to play core roles in the interaction with Plg and Fn [46]. *Mycoplasma conjunctivae* LppT was shown to contain an RGD (Arg-Gly-Asp) motif that is a specific binding site for both Fn and beta heparins of eukaryotic host cells [47]. Through sequence analysis, no RGD motif was found in the MSNOX amino sequence, but two lysine residues were found at the C-terminal (amino acids 455 and 457). However, whether these two lysine residues at the C-terminal of MSNOX participate in the plg- or Fn-binding still needs further exploration.

Owing to the widespread prevalence of MS infection and its substantial economic effects on the chicken industry, establishing an accurate and effective diagnostic method is particularly important. For detection of MS antibodies, the major membrane protein MSPB has been used as a coating antigen, and has been thought to be a specific and sensitive diagnostic antigen [48, 49]. However, MSPB contains a proline-rich repeat region that is prone to insertion or deletion mutations, thus resulting in antigenic variation [50, 51]. Therefore, screening a sensitive, specific, and highly conserved antigen is important. In western blot assays, rMSNOX strongly reacted with the chicken sera positive for various MS isolates (including MS WVU_1853_, JS1, HB1, SD1, and SH1) at 53 kDa, but not with chicken sera positive for MG isolates or several other major avian pathogens. These results confirmed that NOX is a conserved specific immunoreactive protein in MS species. Furthermore, ELISAs also confirmed that rMSNOX had good immunoreactivity and specificity. This study reports the first evidence that NOX protein has potential as a diagnostic antigenic target for MS antibody detection. However, further studies remain necessary.

Complement mediated serum bactericidal activity is mainly activated by forming antigen–antibody complex between antiserum and bacteria, then binding the complement protein C1q. The activated complement system generates opsonic components facilitating phagocytosis of bacteria, which is called classical pathway [52]. The serum bactericidal antibody (SBA) assay which measures complement mediated killing via antibody has been thought as a useful tool for measuring the ability of vaccine-induced antibody to kill *Neisseria meningitidis* or Salmonella [53, 54]. In this study, immunogenicity analysis indicated that rMSNOX is an immunogenic antigen and antiserum against rMSNOX had a significant mycoplasmacidal activity for killing both MS and MG in the presence of complement, indicating the MSNOX may be further studied as a potential protective vaccine candidate. Although MSNOX has been identified as a specific antigen for MS antibody detection, its amino acid sequence shares about 49% homology with that of MGNOX, which may include antigen–antibody binding sites of MG and anti-rMSNOX serum in complement mediated bactericidal activity, thus contribute its significant mycoplasmacidal activity for killing both MS and MG. It has been reported that the mice immunized with rNOX elicit a protective immune response to intranasal or intraperitoneal *S. pneumoniae* challenge, thus suggesting that NOX may be a candidate for a future pneumococcal vaccine [28]. Whether the rMSNOX can be used as a vaccine candidate for protection of MS or MG infection requires further study.

## Conclusion

The rMSNOX was confirmed to be a surface-exposed immunogenic protein that binds DF-1 cells and ECM proteins, including cPlg and hFn. In addition, rabbit anti-rMSNOX serum significantly inhibited the adherence of rMSNOX to DF-1 cells and effectively inhibited the adherence of various MS isolates, but not MG R_low_, to DF-1 cells. Furthermore, the good immunoreactivity and specificity of rMSNOX with MS-positive sera generated by various strains and other sera suggest that it may be used as a candidate diagnostic antigen in the future. Besides, rabbit anti-rMSNOX serum presented complement-dependent mycoplasmacidal activity toward both MS and MG, indicating the MSNOX may be further studied as a potential protective vaccine candidate. This study advances understanding of the biological function of MSNOX protein and its role in the pathogenesis of MS.

## Methods

### Bacterial strains, plasmids, cell lines, and chicken sera positive for various avian pathogens

MS WVU_1853_, MG _Rlow_, and MI 695 were purchased from the China Veterinary Culture Collection Center (CVCC, Beijing, China). MS JS1 and SD1 were isolated from the swollen joints of two diseased chickens from Jiangsu and Shandong provinces in China. MS SH1 and HB1 were isolated from the throat swabs of two diseased chickens from Shanghai and Hubei provinces in China. MG 08, 013, FBH, SGN, and SS were donated by Professor Zhaofeng Sui's research group at Shandong Animal Science and Veterinary College. All strains of MS, MG, and MI were cultured in Mycoplasma Broth Base (Hopebio, China) supplemented with 0.01% nicotinamide adenine dinucleotide (NAD) (Roche, China) and 10% porcine serum (Gibco, USA) at 37 °C in an atmosphere containing 5% CO_2_. Contiguous cell lines of chicken embryo fibroblasts DF-1 were purchased from Shanghai Institute of Biochemistry and Cell Biology and cultured in DMEM (Gibco) supplemented with 10% fetal bovine serum (Gibco) at 37 °C in an atmosphere containing 5% CO_2_. Chicken sera positive for MS strains (including WVU_1853_, JS1, HB1, SD1, and SH1), MI 695, and various MG strains (including R_low_, 08, 013, FBH, MG SGN, and SS) were prepared in our laboratory as described previously [55]. Briefly, cultured MS, MG, and MI were collected and inactivated with 0.4% formaldehyde for 24 h, suspended in phosphate buffered saline (PBS), and emulsified with MONTANIDE ISA 71 VG adjuvant (SEPPIC, France) in a ratio of 3:7, then inoculated into 10-day-old SPF chickens [10^9^ color change units (CCUs) per chicken] subcutaneously two times at 2-week intervals. Two weeks after the second immunization, blood samples were collected to separate antisera. The other chicken sera positive for *E. coli* O1/O2/O78, SPG, PM, STA, NDV, IBDV, and IBV were all obtained from CVCC. The field MS-negative chicken sera (FN-1, FN-2, and FN-3) were collected from a poultry farm in Shanghai, China, and were identified by a MS-antibody ELISA test kit (IDEXX, USA).

### Expression and purification of rMSNOX and rMSFBA

Fructose-1,6-bisphosphate aldolase (FBA) of MS was previously identified as a cytoplasmic protein [56] and was used as a cytoplasmic protein control in this research. MS*nox* and MS*fba* gene fragments were amplified from MS WVU_1853_ by overlap PCR with the primers shown in Table S1, which have been described in our previous studies [26, 56]. The MS*nox* and MS*fba* fragments were ligated into pET28a ( +) (Novagen, USA), and the recombinant strain *E. coli* BL21 (pET28a-MS*nox*) and *E. coli* BL21 (pET28a-MS*fba*) were constr0-ucted. Then the His-tagged rMSNOX protein and rMSFBA protein were expressed and purified as described previously [26, 56]. The purified rMSNOX protein and rMSFBA protein were analyzed with 12.5% SDS-PAGE, stained with Coomassie blue-G250 (Solarbio, China), and imaged with an infrared laser scanning imaging system (Ddyssey; LI-COR, USA). The protein concentrations were detected with a BCA protein assay kit (Beyotime, China).

### Preparation of rabbit antisera

Two-month-old New Zealand white rabbits were purchased from Songlian Experimental Animal Farm (Shanghai, China), and pre-immune serum was collected as a negative control. To prepare polyclonal antibodies against rMSNOX or rMSFBA, we injected rabbits subcutaneously three times at 2-week intervals with 300 µg of purified rMSNOX or rMSFBA protein mixed with an equal volume of Freund’s adjuvant (Sigma, USA). Complete Freund’s adjuvant was used for the first immunization, and incomplete Freund’s adjuvant was used subsequently. Two weeks after the third immunization, blood samples from immunized rabbits were collected to separate antisera. As described above, rabbit sera against MS or MG were also prepared by immunization of rabbits with 10^10^ CCU of inactivated MS WVU_1853_ or MG R_low_ whole cells (incubated with 0.4% formaldehyde for 24 h). The antibody titers of the rabbit sera were analyzed with ELISAs using plates coated with purified rMSNOX protein, purified rMSFBA protein, or whole cell proteins of MS WVU_1853_ or MG R_low_ (0.5 µg/well for each protein). Briefly, 96-well ELISA plates (Corning, USA) were coated with 0.5 μg of rMSNOX, rMSFBA, MS or MG whole cell proteins in carbonate coating buffer (2.94 g/L NaHCO_3_, and 1.6 g/L Na_2_CO_3_, pH 9.6) at 37 °C for 2 h. After being washed three times with PBST, the plates were blocked with 5% non-fat milk in PBST and incubated with serially diluted rabbit antiserum (from 1:100 to 1:204,800) at 37 °C for 1.5 h, then incubated with horseradish peroxidase (HRP)-conjugated goat anti-rabbit IgG (diluted 1:5000; Thermo, USA) at 37 °C for 1 h. In each well,100 μL of soluble TMB substrate solution (Tiangen, China) was added and incubated for 15 min for color reaction, which was then stopped by 50 μL of 2 M H_2_SO_4_. Finally, the absorbance values at 450 nm (OD_450 nm_) were measured with a multi-mode microplatereader (SynergyH1; Biotek, USA). The experiments were performed in triplicate. When the ratio of the OD_450 nm_ value of the antiserum and the pre-immune serum was greater than 2.1, the maximum dilution was the antibody titer of the antiserum.

### Immunogenicity, immunoreactivity and specificity analysis of rMSNOX

Purified rMSNOX and rMSFBA (His-tagged) protein (0.5 µg/well) was subjected to 12.5% SDS-PAGE with PageRuler Prestained Protein Ladder (#26,616, Thermo) and transferred to a nitrocellulose filter (NC) membrane (Amersham, USA). The NC membrane was then blocked with 5% skimmed milk at 37 °C for 2 h and incubated with rabbit anti-rMSNOX serum (1:1000) or pre-immune rabbit serum (1:1000) at 37 °C for 1.5 h. After being washed three times with PBST (PBS adding 0.05% Tween-20), the NC membranes were incubated with HRP-conjugated goat anti-rabbit IgG antibody (1:5000; Thermo) at 37 °C for 1 h. Then the membranes were visualized with a Basic Luminol-enhanced Chemiluminescence (ECL) kit (Yeasen, China) and imaged with a chemiluminescence imager (Tanon 5200; Tanon, China).

To evaluate the immunoreactivity and specificity of rMSNOX with various chicken sera, we performed western blots and ELISAs. In western blot assays, the rMSNOX proteins (0.5 µg/well) were used to react with sera positive for various MS strains (including MS WVU_1853_, JS1, HB1, SD1, and SH1; 1:500) for immunoreactivity detection, and chicken sera against other avian pathogens for specificity analysis, including sera positive for various MG strains (R_low_, 08, 013, FBH, SGN, SS), *E. coli* O1/O2/O78, SPG, PM, STA, NDV, IBDV, and IBV (each diluted 1:500). Three field MS-negative sera (FN-1, FN-2, and FN-3) and SPF chicken serum (CVCC; 1:500) were used as the negative controls. In ELISAs, the procedure was similar to that described above. The 96-well ELISA plates were coated with purified rMSNOX protein (0.5 μg/well), then incubated with chicken sera against various avian pathogens, including all chicken sera used above. After being washed, the plates were incubated with goat anti-chicken IgY-HRP antibody (1:5000; Abbkine, USA), followed by soluble TMB substrate solution (Tiangen); the reaction was stopped with 2 M H_2_SO_4_. Finally, the OD_450 nm_ values were measured as described above.

### Complement dependent mycoplasmacidal assays

Mycoplasmacidal assays were performed as described previously with some modifications [57]. All rabbit sera were inactivated at 56 °C for 30 min before use. MS WVU_1853_, MS JS1, MS SD1, and MG R_low_ were cultured to mid-logarithmic phase, washed three times with PBS, centrifuged at 5000 g for 15 min at 4 °C, and resuspended with PBS. The reaction mixtures containing 30 µL of MS or MG bacterial suspension (7 × 10^8^ CCU/mL), 10 µL of rabbit anti-rMSNOX, anti-MS/MG serum, or pre-immune rabbit serum, and 10 µL of complement (CVCC) were mixed thoroughly and incubated at 37 °C for 1 h. In addition, the reaction mixtures with 10 µL of PBS instead of complement added were also tested as described above and were considered complement-free controls. Finally, each reaction mixture was diluted by tenfold gradient in mycoplasma broth and spread onto solid medium for colony counting. Rabbit anti-MS/MG serum and pre-immune rabbit serum were considered as positive and negative controls, respectively. Three independent experiments were repeated, and the mycoplasmacidal rates were calculated according to the following formula: [(CFU of pre-immune serum treatment–CFU of antiserum treatment)/(CFU of pre-immune serum treatment)].

### Suspension immunofluorescence assays

NOX has been identified to be distributed in both the cytoplasm and cell membrane components of MS in our previous study, according to western blotting assays [26]. To determine the surface localization of NOX on MS, we performed suspension immunofluorescenceassays as previously described [57]. The MS WVU_1853_ was cultured to mid-logarithmic phase, collected by centrifugation at 5000 g, and washed twice with PBS. The MS cells were then fixed with 4% paraformaldehyde at room temperature for 20 min and washed three times with PBS. The fixed MS cells were re-suspended and incubated with rabbit anti-rMSNOX serum (1:1000 diluted by PBS) at 37 °C for 1 h. The rabbit anti-MS serum (1:1000) was used as a positive control, the rabbit anti-rMSFBA serum and pre-immune rabbit serum (1:1000) were used as negative controls. After being washed three times with PBST, the MS cells were incubated with FITC-conjugate goat anti-rabbit IgG (1:1000, Sigma-Aldrich) at 37 °C for 2 h. After being washed, the MS pellets were spread onto glass slides and observed under a fluorescence microscope (Ni-U; Nikon, Japan).

### Immunogold transmission electron microscopy assays

The collected MS cells were fixed with 4% paraformaldehyde and 0.05% glutaraldehyde at room temperature for 2 h, then washed three times with PBS. The fixed MS cells were dehydrated with various concentrations (30, 50, and 70%) of ethanol, and embedded in LR White resin (Sigma, USA). Grids with ultrathin sections were blocked with 5% BSA and then incubated with rabbit anti-MSNOX, anti-rMSFBA serum, or pre-immune rabbit serum (1:1000 diluted by PBST) at 37 °C for 1.5 h. After being washed five times with PBST, the sections were incubated with goat anti-rabbit IgG (whole molecule)-gold antibody (1:100 diluted by PBST; Sigma) at 37 °C for 1 h. After being washed with PBST, the sections were fixed with 2.5% glutaraldehyde for 10 min and stained with uranyl acetate for 5 min and lead citrate for 2 min at room temperature. The dried sections were then visualized with a transmission electron microscope (Tecnai G2 Spirit Biotwin; FEI, USA).

### Adherence and inhibition of adherence of rMSNOX to DF-1 cells

To detect the adherence and inhibition of adherence of rMSNOX to DF-1 cells, we performed indirect immunofluorescence assays as described previously, with some modifications [57]. DF-1 cells were propagated on coverslips in 12-well cell culture plates (Corning) for 24 h. After being washed, the DF-1 cells were incubated with 10 µg of freshly purified rMSNOX in 500 µL DMEM for 1 h at 37 °C. The DF-1 cells adhered by His-tagged rMSFBA protein or no protein were used as controls. For assays of inhibition of adherence, 10 µg of freshly prepared rMSNOX was pre-incubated with rabbit anti-rMSNOX serum (1:50), rabbit anti-MG serum (1:50), or pre-immune rabbit serum (1:50) respectively, at 37 °C for 1 h. Then the serum-treated rMSNOX protein was added to the DF-1 cells for incubation as described above. After incubation, cells were washed four times with PBST to remove the unadhered protein, the bound rMSNOX or rMSFBA protein was recognized by rabbit anti-rMSNOX or rabbit anti-rMSFBA serum (1:1000) for 1 h, and then labeled with goat anti-rabbit IgG-FITC (1:1000; Sigma) for 1 h. The cell membranes and nuclei were stained with 1,1’-dioctadecyl-3,3,3’,3’-tetramethylindodicarbocyanineperchlorate (Dil, Beyotime) and 4’,6-diamidino-2-phenylindole (DAPI, Beyotime), according to methods described previously [57]. Finally, the cellular coverslips were treated with antifade mounting medium (Sangon Biotech, China) and observed under a laser scanning confocal microscope (LSM800; Zeiss, German). The experiments were performed in triplicate. The FI of FITC and DAPI was quantitatively assessed by ImageJ software and the FI ratios of FITC to DAPI were analyzed.

### Adherence and inhibition of adherence of MS/MG to DF-1 cells

The inhibition of adherence of MS or MG to DF-1 cells by anti-rMSNOX serum was estimated with indirect immunofluorescence and colony counting assays. DF-1 cells were propagated in DMEM on microscope coverslips in six-well cell culture plates (Corning) for 24 h. MS WVU_1853_, MS JS1, MS SD1, and MG R_low_ were cultured to logarithmic growth phase and collected by centrifugation at 5000 g for 15 min. Then the three MS isolates and MG R_low_ (2 × 10^8^ CCU/mL) were incubated with rabbit anti-rMSNOX, anti-MS (for MS isolates only), anti-MG (for MG R_low_ only), or pre-immune serum (1:50) at 37 °C for 1 h. The rabbit anti-MS/MG and pre-immune serum were used as positive and negative controls. All rabbit sera were inactivated at 56 °C before use. The DF-1 cells were infected with pre-treated MS or MG cells (6 × 10^7^ CCU/well) at a multiplicity of infection of 200 at 37 °C for 2 h, then were washed thoroughly with PBS to remove the un-adhered mycoplasma. For indirect immunofluorescence assays, adhered mycoplasma cells were recognized by rabbit anti-MS or anti-MG serum (1:1000) for 1 h, and were then labeled with goat anti-rabbit IgG-FITC (1:1000, Sigma) for 1 h. The DF-1 cell membranes and nuclei were stained with Dil (1:100, Beyotime) and DAPI (1:1000, Beyotime) and observed under a fluorescence microscope (Ni-U; Nikon). The FIs of FITC and DAPI from three independent experiments were quantitatively assessed by ImageJ software and the FI ratios of FITC to DAPI were analyzed. For colony counting assays, the DF-1 cells were scraped and then lysed in 1 mL serum-free mycoplasma culture medium for 20 min. The suspension was serially diluted and spread onto mycoplasma agar plates for colony counting. Three independent experiments were performed in triplicate. The inhibition of adherence rate was calculated as [(CFU from pre-immune serum treatment–CFU from antiserum treatment)/CFU from pre-immune serum treatment] × 100%.

### Binding activity of rMSNOX to cPlg and hFn

Western blotting and ELISA were used to determine the binding activity of rMSNOX to cPlg and hFn.

For western blot analysis, gradient diluted rMSNOX protein (2, 1, and 0.5 µg) and 2 µg BSA (Sigma) were subjected to 12.5% SDS-PAGE, then transferred to an NC membrane. After blocking with 5% skimmed milk, the NC membrane was incubated with 10 µg/mL of cPlg (Cell Sciences, USA) or hFn (Sigma) for 2 h at 37 °C. After excessive washing with PBST, the membrane was incubated with rabbit anti-cPlg/hFn polyclonal antibody (1:1000; Cell Sciences) for 1 h at 37 °C, then incubated with HRP conjugated goat anti-rabbit IgG antibody (1:5000; Thermo) for 1 h at 37 °C. Membranes were visualized with a Basic Luminol ECL kit (Yeasen).

For ELISA analysis, the 96-well plates (Corning) were coated with 1 µg/well of cPlg, hFn or BSA (negative control). After blocking with 5% skimmed milk, wells were incubated with various amounts of rMSNOX protein (1, 0.5, 0.15, 0.125, 0.0625, and 0.03125 µg/well) at 37 °C for 1.5 h. After being washed, the wells were treated with rabbit anti-rMSNOX serum (1:500) at 37 °C for 1 h, followed by HRP conjugated goat anti-rabbit IgG antibody (1:5000; Thermo) at 37 °C for 1 h. TMB substrate solution and 2 M H_2_SO_4_ were added successively and OD_450 nm_ values were measured as described above. The experiments were performed in triplicate.

### Statistical analysis

Data are given as the mean with standard deviation for three replicate experiments, and statistical analyses were performed with unpaired T-test and one/two-way ANOVA in GraphPad Prism6 software. Significant differences are denoted ^***^p < 0.001 or ^****^p < 0.0001, and ns represents no significance.

## Supplementary Information

**Additional file 1:** **Table S1.** Primers used for overlap PCR amplification of MS*nox* [26] and MS*fba*  [56] genes. **Fig. S1.** Detection of antibody titers of different rabbit antisera by ELISA. The ELISA plates were coated with 0.5 μg of rMSNOX , rMSFBA, MS whole cell proteins or MG whole cell proteins respectively. Then the plates were reacted with serially diluted rabbit anti-rMSNOX, anti-rMSFBA, anti-MS or anti-MG serum, respectively. When the ratios of the OD_450nm_ value of the antiserum and the pre-immune serum (marked above the black column graph) was greater than 2.1, the maximum dilution was determined as the antibody titer of the antiserum. **Fig. S2.** Full-length figure for expression and purification of rMSNOX and rMSFBA protein. Lane M: protein marker; lane 1 and 5: cell lysates of *E. coli* BL21 containing empty vector; lane 2 and 6: total cell lysates of recombinant strain *E. coli* BL21 (pET28a-MS*nox*) and *E. coli* BL21 (pET28a-MS*nox*); lane 3 and 7: supernatant of total cell lysates of recombinant bacteria* E. coli* BL21 (pET28a-MS*nox*) and *E. coli* BL21 (pET28a-MS*nox*); lane 4 and 8: purified His-tagged MSNOX protein and His-tagged MSFBA protein. **Fig. S3.** Original figure for immunogenicity analysis of rMSNOX protein. Lane M: protein marker; lane 1 and 3: purified His-tagged rMSFBA protein (His-tag control) reacted with rabbit anti-rMSNOX serum and pre-immune rabbit serum (1:1000), respectively; lane 2 and 4: purified His-tagged MSNOX rabbit reacted with rabbit anti-rMSNOX serum and pre-immune rabbit serum (1:1000), respectively. **Fig. S4.** Full-length blots for reactivity analysis of rMSNOX with different chicken sera. Purified rMSNOX protein (0.5 μg/well) was subjected to 12.5% SDS-PAGE followed by transferring to NC membrane. The NC membranes were cut to react with different chicken sera, including positive chicken serum against different MS isolates (MS WVU_1853_, MS JS1, MS HB1, MS SD1 and MS SH1), positive chicken sera of different MG isolates (MG R_low_, MG 08, MG 013, MG FBH, MG SGN and MG SS), positive sera against other avian pathogens (MI, *E. coli* O1/O2/O78, SPG, PM, STA, NDV, IBDV and IBV), three field MS-negative sera (FN-1, FN-2 and FN-3), and negative serum from SPF chicken. All of the chicken sera were diluted at 1:500. For Western blots using positive chicken serum against PM and STA, whole cell proteins of PM or STA were in lane 1 respectively, and 0.5 μg of rMSNOX protein were in lane 2. **Fig. S5.** Full-length figures for cPlg and hFn binding assays using Western blots. Serially diluted rMSNOX protein (lanes 1-3: 2, 1, and 0.5 μg) and 2 μg BSA (lane 4) were incubated with 10 μg/mL of cPlg (A) or hFn (B), and then recognized by rabbit anti-cPlg or anti-hFn polyclonal antibody (1:1000).  

## Data Availability

All data generated or analysed during this study are included in this published article. Further inquiries can be directed to the corresponding authors.

## Abbreviations

MS: *Mycoplasma synoviae*
NOX: NADH oxidase
MSNOX: NOX of *Mycoplasma synoviae*
rMSNOX: Recombinant MSNOX
Plg: Plasminogen
Fn: Fibronectin
ECM: Extracellular matrix
VlhA: Variable lipoprotein hemagglutinin
GAPDH: Glyceraldehyde-3-phosphate dehydrogenase
PdhA and PdhB: Pyruvate dehydrogenase alpha and beta subunits
*M.bovis*: *Mycoplasma bovis*
SDS-PAGE: Sodium dodecyl sulfate polyacrylamide gel electrophoresis
SPF: Specific pathogen free
MG: *Mycoplasma gallisepticum*
*E. coli*: *Escherichia coli*
SPG: *Salmonella pullorum*/*gallinarum*
PM: *Pasteurella multocida*
STA: *Staphylococcus aureus*
NDV: Newcastle disease virus
IBDV: Infectious bursal disease virus
IBV: Avian infectious bronchitis virus
FITC: Fluorescein isothiocyanate
DMEM: Dulbecco’s modified eagle medium
cPlg: Chicken plasminogen
hFn: Human fibronectin
BSA: Bovine serum albumin
*S. pneumoniae*: *Streptococcus pneumoniae*
NAD: Nicotinamide adenine dinucleotide
CCU: Color change unit
NC: Nitrocellulose filter
HRP: Horseradish peroxidase
ECL: Enhanced chemiluminescence
CFU: Colony-forming units
PBS: Phosphate buffer saline
FBA: Fructose-1,6-bisphosphate aldolase
Dil: 1,1’-Dioctadecyl-3,3,3’,3’-tetramethylindodicarbocyanine perchlorate
DAPI: 4’,6-Diamidino-2-phenylindole
MOI: Multiplicity of infection
SD: Standard deviation
FI: Fluorescence intensity
