# Supplementary material for: NADH oxidase of Mycoplasma synoviae is a potential diagnostic antigen, plasminogen/fibronectin binding protein and a putative adhesin
Source: BMC Vet Res. 2022 Dec 29;18:455. doi: 10.1186/s12917-022-03556-2 (PMC9798693; doi:10.1186/s12917-022-03556-2)
Supplement: Supplementary file 1 — Additional file 1: Table S1. Primers used for overlap PCR amplification of MSnox [26] and MSfba [56] genes. Fig. S1. Detection of antibody titers of different rabbit antisera by ELISA. The ELISA plates were coated with 0.5 μg of rMSNOX , rMSFBA, MS whole cell proteins or MG whole cell proteins respectively. Then the plates were reacted with serially diluted rabbit anti-rMSNOX, anti-rMSFBA, anti-MS or anti-MG serum, respectively. When the ratios of the OD450nm value of the antiserum and the pre-immune serum (marked above the black column graph) was greater than 2.1, the maximum dilution was determined as the antibody titer of the antiserum. Fig. S2. Full-length figure for expression and purification of rMSNOX and rMSFBA protein. Lane M: protein marker; lane 1 and 5: cell lysates of E. coli BL21 containing empty vector; lane 2 and 6: total cell lysates of recombinant strain E. coli BL21 (pET28a-MSnox) and E. coli BL21 (pET28a-MSnox); lane 3 and 7: supernatant of total cell lysates of recombinant bacteria E. coli BL21 (pET28a-MSnox) and E. coli BL21 (pET28a-MSnox); lane 4 and 8: purified His-tagged MSNOX protein and His-tagged MSFBA protein. Fig. S3. Original figure for immunogenicity analysis of rMSNOX protein. Lane M: protein marker; lane 1 and 3: purified His-tagged rMSFBA protein (His-tag control) reacted with rabbit anti-rMSNOX serum and pre-immune rabbit serum (1:1000), respectively; lane 2 and 4: purified His-tagged MSNOX rabbit reacted with rabbit anti-rMSNOX serum and pre-immune rabbit serum (1:1000), respectively. Fig. S4. Full-length blots for reactivity analysis of rMSNOX with different chicken sera. Purified rMSNOX protein (0.5 μg/well) was subjected to 12.5% SDS-PAGE followed by transferring to NC membrane. The NC membranes were cut to react with different chicken sera, including positive chicken serum against different MS isolates (MS WVU1853, MS JS1, MS HB1, MS SD1 and MS SH1), positive chicken sera of different MG isolates (MG Rlow, MG 08, MG 013, MG F [file 12917_2022_3556_MOESM1_ESM.docx]

**Supplementary tables and figures**

**Table S1 Primers used for overlap PCR amplification of MS*nox* [26] and MS*fba* [56] genes.**

| **Target gnes** | **Primers name** | **Primers sequence(5'→3′)** | **Products (bp)** |
| --- | --- | --- | --- |
|  | MS*nox* 1F | GGATCCATGGAAAACAATAAAATTATAG | 154 |
|  | MS*nox* 1R | AACATTCCTCCAAC*C*CAAACAG |  |
| MS*nox* | MS*nox* 2F | GCTGTTTG*G*GTTGGAGGAATG | 226 |
|  | MS*nox* 2R | ATGGAGGAACTATAGG*C*CATGTTC |  |
|  | MS*nox* 3F | GCTGGAGGAACATG*G*CCTATAG | 869 |
|  | MS*nox* 3R | AATCTTGTCC*C*CAAGAACCAAC |  |
|  | MS*nox* 4F | CAAGTTGGTTCTTG*G*GGACAAG | 182 |
|  | MS*nox* 4R | CTCGAG TTAAGCTTTATATTTTAAACC |  |
|  | MS*fba* 1F | CGGATCCATGCCATTAACAAACG | 106 |
|  | MSf*ba* 1R | GAAGCACAGCTTTAGC*C*CATTCTA |  |
| MS*fba* | MS*fba* 2F | TAGAATG*G*GCTAAAGCTGTGCTTC | 487 |
|  | MS*fba* 2R | TCAAGAGATTT*C*CAATTTTCTGGATATGGC |  |
|  | MS*fba* 3F | GCCATATCCAGAAAATTG*G*AAATCTCTTGA | 328 |
|  | MS*fba* 3R | CGGAATTCTTAAGCTTTATTTTGTGATCC |  |

Note: The restriction enzyme sites of *Bam*H I (GGATCC), *Xho* I (CTCGAG) and *Eco*R I (GAATTC) were underlined; the mutated nucleotides were in italics.

**Fig. S1**

**
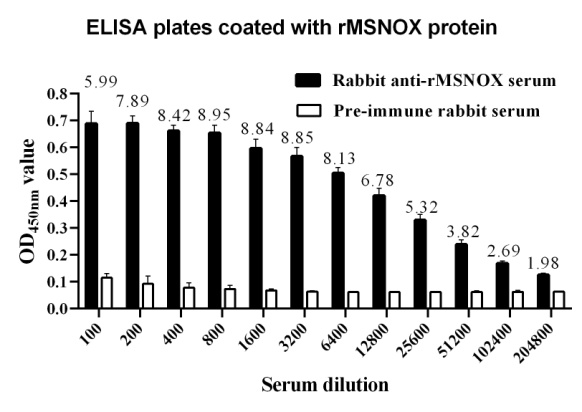

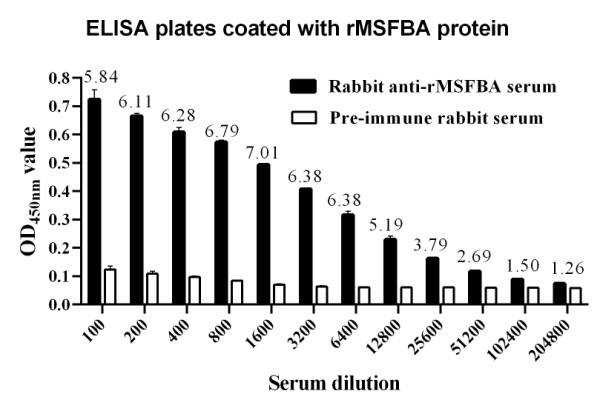
** **
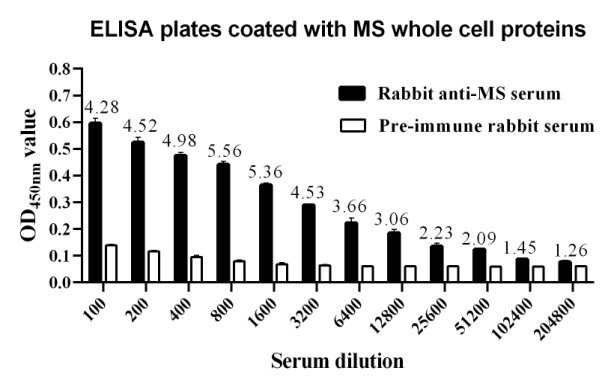

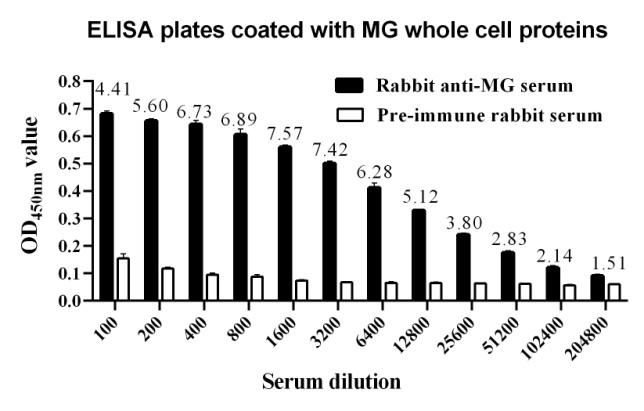
**

**Fig. S1. Detection of antibody titers of different rabbit antisera by ELISA.** The ELISA plates were coated with 0.5 μg of rMSNOX , rMSFBA, MS whole cell proteins or MG whole cell proteins respectively. Then the plates were reacted with serially diluted rabbit anti-rMSNOX, anti-rMSFBA, anti-MS or anti-MG serum, respectively. When the ratios of the OD_450nm_ value of the antiserum and the pre-immune serum (marked above the black column graph) was greater than 2.1, the maximum dilution was determined as the antibody titer of the antiserum.

**Fig. S2**

M 1 2 3 4 M 5 6 7 8

kDa


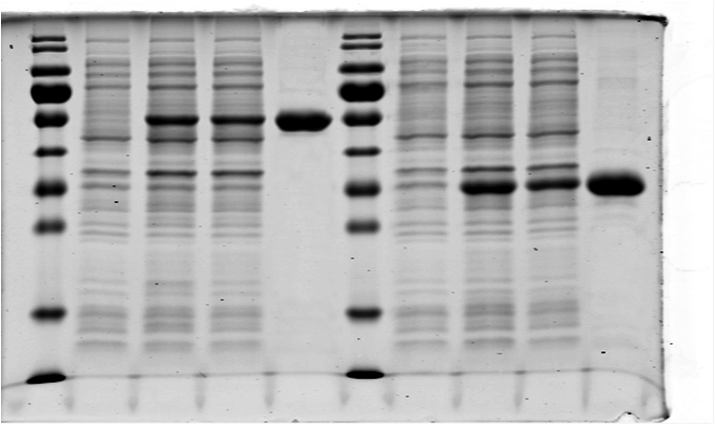


53 kDa

33 kDa

170

130

100

70

55

40

35

25

10

15

**Fig. S2. Full-length figure for expression and purification of rMSNOX and rMSFBA protein.** Lane M: protein marker; lane 1 and 5: cell lysates of *E. coli* BL21 containing empty vector; lane 2 and 6: total cell lysates of recombinant strain *E. coli* BL21 (pET28a-MS*nox*) and *E. coli* BL21 (pET28a-MS*nox*); lane 3 and 7: supernatant of total cell lysates of recombinant bacteria *E. coli* BL21 (pET28a-MS*nox*) and *E. coli* BL21 (pET28a-MS*nox*); lane 4 and 8: purified His-tagged MSNOX protein and His-tagged MSFBA protein.

**Fig. S3**


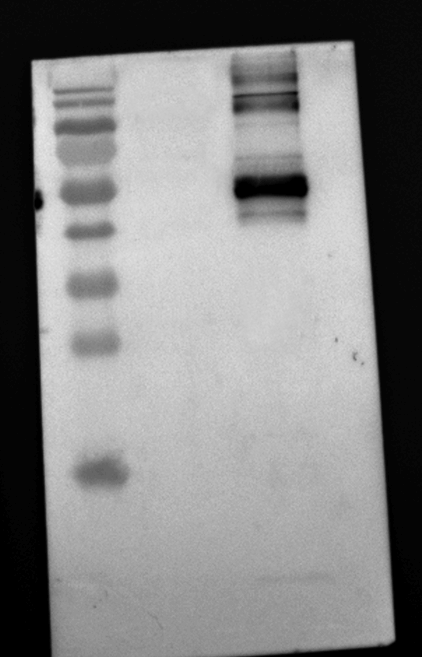

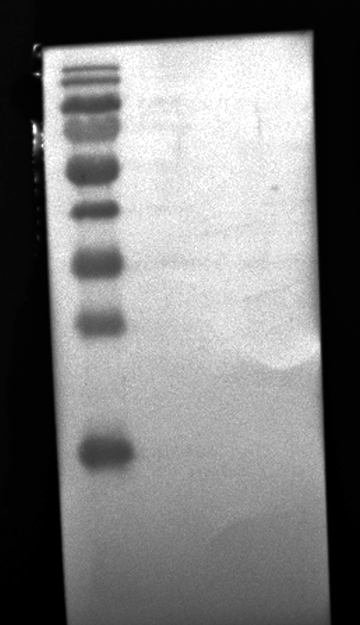


kDa

40

55

53 kDa

M 1 2 M 3 4

**Fig. S3. Original figure for immunogenicity analysis of rMSNOX protein.** Lane M: protein marker; lane 1 and 3: purified His-tagged rMSFBA protein (His-tag control) reacted with rabbit anti-rMSNOX serum and pre-immune rabbit serum (1:1000), respectively; lane 2 and 4: purified His-tagged MSNOX rabbit reacted with rabbit anti-rMSNOX serum and pre-immune rabbit serum (1:1000), respectively.

**Fig.S4**

MS SH1(+)

MS SD1(+)

MS HB1(+)

MS JS1(+)

MS WVU_1853_(+)


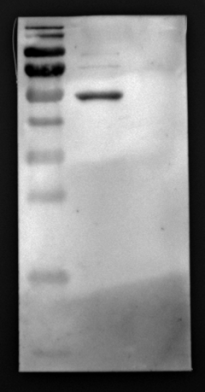

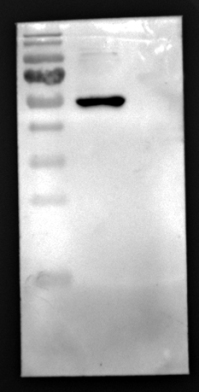

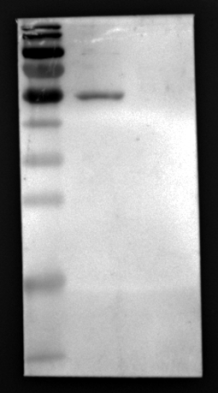

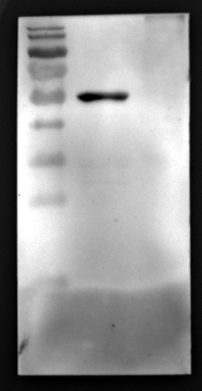

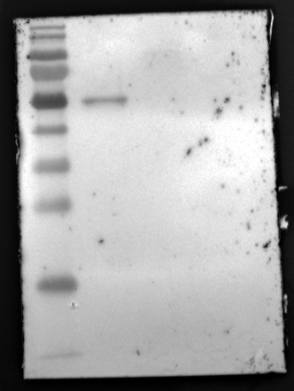


MG SGN (+)

MG FBH (+)

MG Rlow (+)

MG 013 (+)

MG 08 (+)


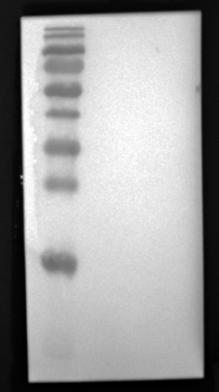

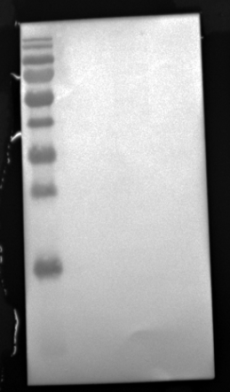

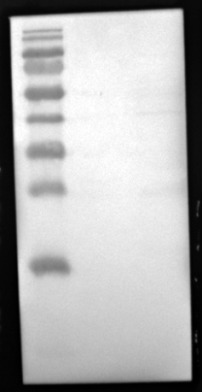

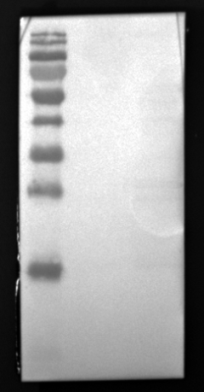

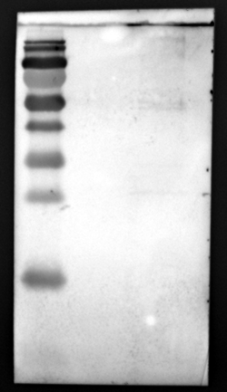


SPG (+)

PM (+)

*E. coli* (+)

MI (+)

MG SS (+)


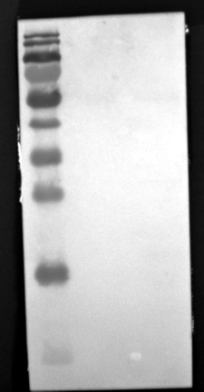

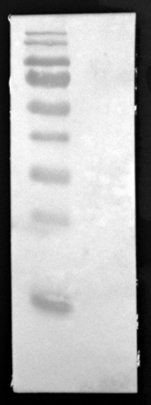

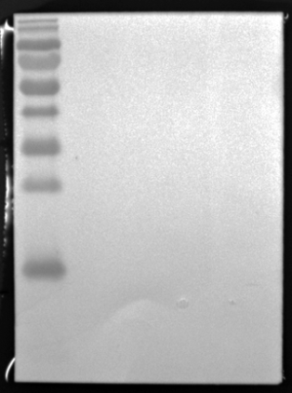

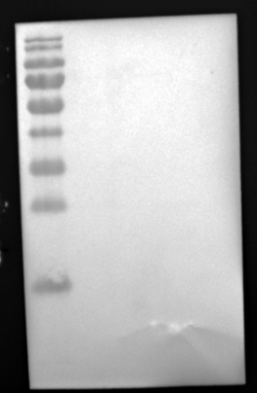

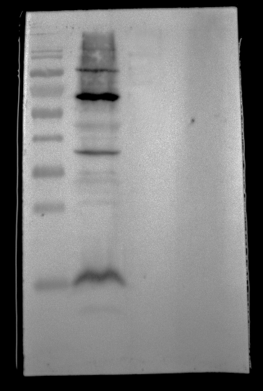


1 2

FN-1

IBV (+)

IBDV (+)

NDV (+)

STA (+)


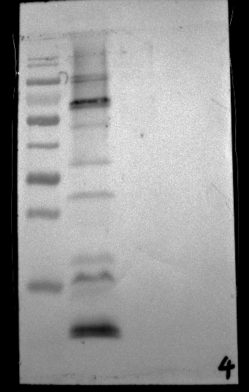

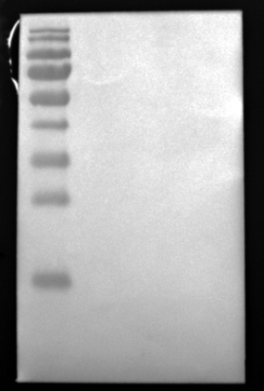

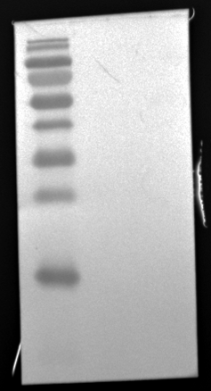

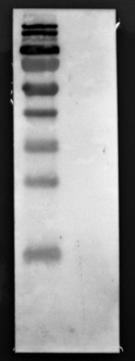

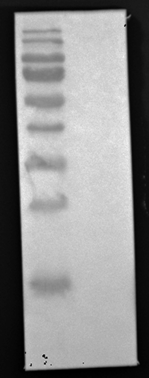


1 2

FN-2

FN-3

SPF


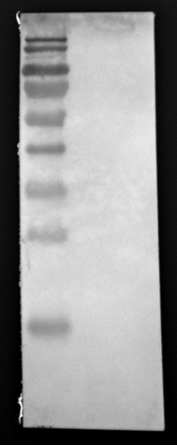

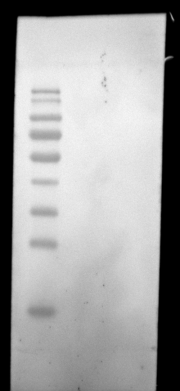

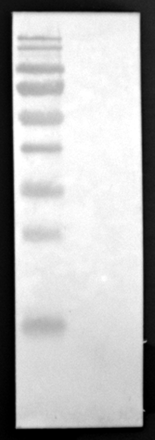


**Fig. S4. Full-length blots for reactivity analysis of rMSNOX with different chicken sera.** Purified rMSNOX protein (0.5 μg/well) was subjected to 12.5% SDS-PAGE followed by transferring to NC membrane. The NC membranes were cut to react with different chicken sera, including positive chicken serum against different MS isolates (MS WVU_1853_, MS JS1, MS HB1, MS SD1 and MS SH1), positive chicken sera of different MG isolates (MG R_low_, MG 08, MG 013, MG FBH, MG SGN and MG SS), positive sera against other avian pathogens (MI, *E. coli* O1/O2/O78, SPG, PM, STA, NDV, IBDV and IBV), three field MS-negative sera (FN-1, FN-2 and FN-3), and negative serum from SPF chicken. All of the chicken sera were diluted at 1:500. For Western blots using positive chicken serum against PM and STA, whole cell proteins of PM or STA were in lane 1 respectively, and 0.5 μg of rMSNOX protein were in lane 2.

**Fig. S5**

**B**

**A**

M 1 2 3 4 M 1 2 3 4


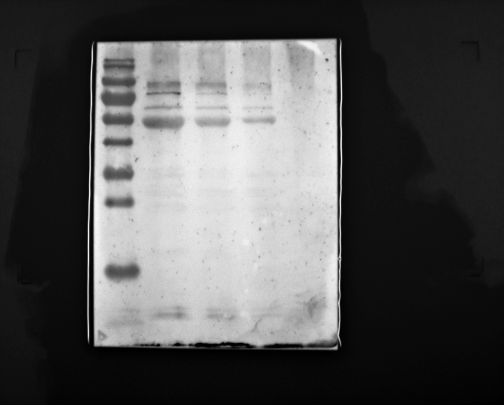

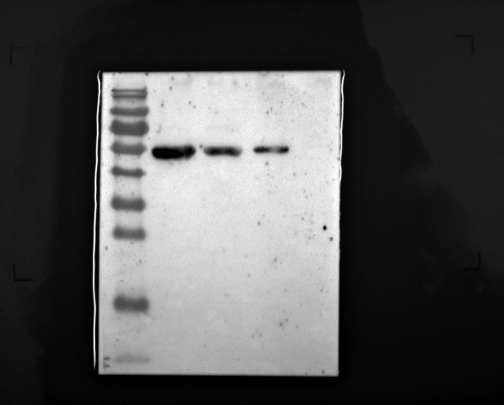


**Fig. S5. Full-length figures for cPlg and hFn binding assays using Western blots.** Serially diluted rMSNOX protein (lanes 1-3: 2, 1, and 0.5 μg) and 2 μg BSA (lane 4) were incubated with 10 μg/mL of cPlg (A) or hFn (B), and then recognized by rabbit anti-cPlg or anti-hFn polyclonal antibody (1:1000).
